# Editorial: Co-designing and evaluating oral health promotion interventions for vulnerable groups

**DOI:** 10.3389/froh.2025.1587771

**Published:** 2025-03-21

**Authors:** Vanessa Muirhead, Maha El Tantawi, Andrea Rodriguez

**Affiliations:** ^1^ Institute of Dentistry, Queen Mary University of London, London, United Kingdom; ^2^ Faculty of Dentistry, Alexandria University, Alexandria, Egypt; ^3^ School of Dentistry, University of Dundee, Dundee, United Kingdom

**Keywords:** co-design, health promotion, vulnerable children, participatory research, diversity

**Editorial on the Research Topic**
Co-designing and evaluating oral health promotion interventions for vulnerable groups

This Research Topic (RT) has attracted authors who have engaged with underrepresented groups in oral health and health promotion research. These authors have worked “with”, rather than “on” people who are perceived as vulnerable using qualitative and participatory research, public engagement and health interventions to reduce social exclusion and inequities. The nine papers demonstrate the diversity of participatory approaches (1) at different stages of the research process, such as co-creation, co-design, and co-production.

The authors reported on research with diverse groups. Høiseth and Jasbi and Jasbi et al. engaged with adolescents and public dental services to understand adolescents' perspectives on oral health care and promotion, and to explore innovative techniques for dental professionals to promote hope. Booth et al. engaged with ex-offenders and third-sector organisations to co-design a film showcasing the dental experiences of this group before and after their transition out of prison. Cairns and Rodriguez involved “experts by experience” and their health and social care providers to co-design a dental service for adults experiencing homelessness. Chauhan et al. engaged with parents of young children in high caries communities who had limited proficiency in English to explore the accessibility of the “HABIT” intervention. Paisi et al. engaged with a range of participants that included dental and healthcare professionals, peer researchers, community representatives, patients, and support workers to co-design, implement and evaluate a dental service for people experiencing Severe and Multiple Disadvantages in England. Rodriguez et al. scoping review identified literature on the participation of people experiencing homelessness and/or their support workers in co-designing health and oral health promotion materials. Doughty et al. involved people living with HIV, those experiencing homelessness and those who identify as heterosexual in a study that demonstrated how Public and Patient Involvement was embedded in the development of an HIV testing intervention for dental settings. Beaton et al. engaged with oral health practitioners from a national oral health improvement programme in Scotland that evaluated the influence of the Smile4life intervention on the engagement behaviours of practitioners.

Høiseth and Jasbi illustrated the early co-creation that shaped the research questions and design of tailored oral health promotion for adolescents in the #Care4YoungTeeth<3 programme. This clearly required extensive collaboration among oral health professionals, designers and digital storytelling specialists.

Jasbi et al. highlighted the need for multidisciplinary collaboration and equal participation throughout the research process to foster adolescent empowerment during dental consultations. In this way, tailored co-designed interventions were able to transform challenges such as anxiety into positive dental experiences.

Booth et al. illustrated how they actively involved ex-offenders by using films to depict the stigma of people who experienced the justice system. Their approach dispelled the power differentials typically found in traditional researcher-led studies and facilitated inclusive collaboration.

Cairns and Rodriguez used the co-design framework for healthcare innovation to co-design a dental service for adults experiencing homelessness in a city with a high level of homelessness in Scotland.

Chauhan et al. used co-production at the latter stage of their research to inform strategies to improve the uptake of oral health resources. Participants with limited English proficiency described how they used translation tools, sought support from family and friends and recommended including visuals to increase understanding. The authors used this feedback to modify their resources.

Several papers highlighted the benefits of participatory approaches in intervention development for underserved communities. Paisi et al. described how they co-designed, implemented and evaluated a new dental service for people experiencing severe and multiple disadvantages. They emphasised the need for collaborative working, flexibility and support for people managing complex and chaotic lifestyles, and education for the dental workforce in trauma-informed dental practice.

This RT addressed the real-world challenges of participatory approaches. Rodriguez et al. scoping review described the barriers and enablers encountered while co-designing educational resources for people experiencing homelessness, such as recruiting, maintaining relationships, power differentials, time constraints and limited resources. Doughty et al. described their learning from involving patients and the public in developing a HIV-testing intervention from the perspective of finding “one's feet” as a novice PhD student and early career researcher. At the opposite end of the spectrum, Beaton et al. explored practitioners' experiences of delivering the national oral health programme Smile4life for people experiencing homelessness. They shared their own experiences of responding to challenging situations and the ability to act as a “boundary spanner” when exposed to a range of opinions, working environments and cultures of homeless organisations.

The nine articles identified key principles of co-design that enhanced the representativeness and inclusiveness of their findings (2). The principles of trust (Cairns and Rodriguez), empowerment, and non-judgemental attitudes (Rodriguez et al.) through working closely with the community, alongside the need to embrace flexibility (Cairns and Rodriguez, Chauhan et al., Paisi et al., Rodriguez et al.) were highlighted by the authors. The need to build a culture of involvement at all stages of the research process (Doughty et al.), by valuing equal opportunities and levels of participation with well-structured channels to listen to and integrate participants' views (Høiseth and Jasbi, Booth et al.) and multidisciplinary collaboration (Jasbi et al.) was also perceived as an important principle. This approach demonstrates the value of adapting research methods to the preferences and needs of the community.

Why do researchers make the conscious decision to pursue participatory approaches despite these challenges and barriers? We argue in this editorial that researchers choose and are inspired to adopt these counter-cultural empowerment research and engagement approaches that elevate the voices and lived experiences of vulnerable and marginalised communities (3) because of the richness, learning and impact that ensue beyond the research and outcomes. “Co” approaches create unexplained freedom for researchers who learn to embrace the inevitable uncertainty of not knowing – navigating the perilous seas outside the researchers' control. Participatory approaches offer rewards through reflexivity and by fostering intellectual humility, which means being open to new ideas and challenging perspectives. This creates space for deeper thinking, flexibility and critical reflection (4).

This editorial ends with a call to action to encourage innovative participatory approaches, creative methodologies, supporting funding streams and the development of a community of practice to promote participatory oral health research.
